# The Journey of iPSC-derived OPCs in Demyelinating Disorders: From *In vitro* Generation to *In vivo* Transplantation

**DOI:** 10.2174/1570159X21666230220150010

**Published:** 2023-07-10

**Authors:** Fatemeh Lohrasbi, Maryam Ghasemi-Kasman, Negar Soghli, Sobhan Ghazvini, Zahra Vaziri, Sadaf Abdi, Yasaman Mahdizadeh Darban

**Affiliations:** 1 Student Research Committee, Babol University of Medical Science, Babol, Iran;; 2 Cellular and Molecular Biology Research Center, Health Research Institute, Babol University of Medical Science, Babol, Iran;; 3 Department of Physiology, School of Medical Sciences, Babol University of Medical Science, Babol, Iran

**Keywords:** Demyelination, stem cells, reprogramming, iPSCs, OPCs, repair

## Abstract

Loss of myelination is common among neurological diseases. It causes significant disability, even death, if it is not treated instantly. Different mechanisms involve the pathophysiology of demyelinating diseases, such as genetic background, infectious, and autoimmune inflammation. Recently, regenerative medicine and stem cell therapy have shown to be promising for the treatment of demyelinating disorders. Stem cells, including embryonic stem cells (ESCs), induced pluripotent stem cells (iPSCs), and adult stem cells (ASCs), can differentiate into oligodendrocyte progenitor cells (OPCs), which may convert to oligodendrocytes (OLs) and recover myelination. IPSCs provide an endless source for OPCs generation. However, the restricted capacity of proliferation, differentiation, migration, and myelination of iPSC-derived OPCs is a notable gap for future studies. In this article, we have first reviewed stem cell therapy in demyelinating diseases. Secondly, methods of different protocols have been discussed among *in vitro* and *in vivo* studies on iPSC-derived OPCs to contrast OPCs’ transplantation efficacy. Lastly, we have reviewed the results of iPSCs-derived OLs production in each demyelination model.

## 1 INTRODUCTION

Demyelinating diseases of the central nervous system (CNS) are a heterogeneous group of disorders involving multiple sclerosis (MS), optic neuritis, neuromyelitis optica (Devic's disease), and acute disseminated encephalomyelitis. MS is a chronic inflammatory disease, being the most frequent demyelinating disease of the CNS [1].

Demyelination could be the result of autoimmune inflammatory demyelinating diseases or the demyelinating disease of genetic origin [2]. In inflammatory demyelinating disease, lymphocytic infiltration harms the myelin and axons of the CNS [3].

Although there is no cure for demyelinating diseases, treatments focus on slowing the disease progression and accelerating the recovery [4].

The major issue in demyelinating diseases is the damaged myelin sheath. It creates demyelination plaques in both gray matter and white matter. While oligodendrocytes (OLs) are effective for remyelination, they are not capable of proliferating or migrating. Moreover, the number of oligodendrocyte progenitor cells (OPCs) should be enhanced [5]. Therefore, stem cell therapy could be beneficial in encouraging the number of OPCs. The discovery of induced pluripotent stem cells (iPSCs) paved the way for a large quantity of autologous OPCs derived from somatic cells. In this technique, adult fully differentiated somatic cells like epithelial cells are converted into pluripotent cells by transcription factors and chemical compounds [6]. The iPSC-derived OPCs can remyelinate the axons but with a low capacity of migration.

This review investigates deployments of iPSC-derived OPCs as a therapeutic approach for the demyelinating disease. After that, animal models, researches on iPSC and OPC, limitations, and advantages of future treatments, are discussed.

## 2 STEM CELLS THERAPY

Stem cells are undifferentiated cells with the potential of self-renewal and differentiating into specific cell types. Due to their potency, they are divided into totipotent, pluripotent, and multipotent stem cells [7, 8]. Totipotent stem cells can differentiate into all types of cells presenting in a living organism, even placenta. Pluripotent stem cells, which exist in the inner layer of blastocyst, can produce in all organs of the body, except for placenta. Multipotent stem cells, which are the result of pluripotent stem cells differentiation, can produce cell types of their lineage [7]. Also, stem cells include embryonic stem cells (ESCs), induced pluripotent stem cells (iPSCs), and adult stem cells (ASCs), based on their origin of formation. ESCs and iPSCs are pluripotent cells, while ASCs include neural stem cells (NSCs), hematopoietic stem cells (HSCs), and mesenchymal stem cells (MSC), which are multipotent [7-9].

ESC-derived OLs can form myelin, but they are restricted because of tumorigenesis, immune response, and moral issues [10]. ASCs include NSCs, MSCs, and HSCs. NSCs are present in small numbers in the sub-ventricular (SVZ) and sub-granular zones (SGZ) of the brain. They can create OLs, but the limitation is the hard isolation and purification [11, 12]. In addition, the inflammatory microenvironment of the lesions affects the quality of migration and differentiation of transplanted NSCs [8]. Mesenchymal stem cells (MSCs) have synaptogenesis and neuroprotective functions [13]. They are more efficient in patients with active lesions who have been diagnosed in the early stages of MS. Therefore, choosing MSC therapy is related to the level of the pathological process of the disease [14]. HSCs transplantation is usually used for patients with malignant MS, but with several limitations.

Autologous HSC transplantation is associated with side effects, including neurotoxicity, transient alopecia, amenorrhea, and secondary autoimmune disease [15].

iPSCs are reprogrammed pluripotent stem cells [8]. iPSC-derived OPCs have a neuroprotective function, and they lead to remyelination [11]. Moreover, the safety concerns of allogenic grafts have disappeared by autologous transplantation of iPSC-derived OPCs [16]. Yamanaka and his colleagues found four genes that could promote pluripotency, including Oct3/4, Sox2, c-Myc, and Klf-4 [17, 18]. Besides the advantages of iPSCs, they may increase the risk of tumorigenesis [8]. However, trans-differentiation is the key process to diminish the possibility of tumorigenesis [19]. Trans-differentiation is a direct conversion of one differentiated cell type into another without passing through the pluripotent phase [20]. Trans-differentiation of exocrine cells of the pancreas into β-cells is a successful example of this technique in regenerative medicine [21]. Trans-differentiation may occur naturally or experimentally. Natural trans-differentiation is based on two processes: first, cells dedifferentiate, and then the reprogramming process takes place. Experimental trans-differentiation may occur with the dedifferentiation of cells to produce intermediate cell types. It can also occur with direct conversion of one cell type into another [22]. Trans-differentiation starts with slow dedifferentiation followed by passing cell cycle to generate the new cell lineage by expression of transcription factors and ceasing the expression of some genes. Also, somatic mutations of the genes are needed for differentiation, and epigenetic changes are essential for trans-differentiation [21].

Current chemical drugs for MS are helpful for anti-inflammatory functions. Drugs may suppress the immune response and limit the demyelination process. However, they cannot effectively induce remyelination of the CNS, while cell therapy has successfully made it happen [13, 23]. Cell therapies are based on two main mechanisms: 1) cell replacement by engraftment; 2) tissue recovery and rebuilding of stem cells by self-renewal process with cytokines and growth factors [9].

## 3 INDUCED PLURIPOTENT STEM CELLS

### 3.1 iPSCs

iPSCs and ESCs are very similar, with some intricate genomic differences; for instance, ESCs contain large numbers of duplications within a small subset of samples, meanwhile iPSCs exhibit moderate numbers of deletions throughout several iPSC lines [24]. At the blastocyst stage, human embryonic stem cells (hESCs) are formed from the inner cell mass of a developing embryo. The differentiation of ESCs would make cells more committed, functionally limited, and unidirectional [24, 25].

### 3.2 Reprogramming

Cell reprogramming is the process of reversing an adult cell to its initial state by repressing the genes responsible for differentiation and activating the relevant genes for reprogramming.

Varied approaches are available for inducing pluripotency, which include Somatic Cell Nuclear Transfer (SCNT), cell fusion, transduction by OSKM transcription factors (direct programming), and reprogramming with small molecules.

#### 3.2.1 Nuclear Transfer

In this technique, the egg cell nucleus must be removed, and then stimulated by a shock that forms blastocyst. In SCNT, electrical or chemical stimulation activates the egg, removes ESCs from the developing blastocyst, and causes the development of embryo. However, studies have not shown enough efficiency of this process.

#### 3.2.2 Cell Fusion

It is a natural process of somatic cells to fuse with pluripotent cells in order to reprogram the somatic cell’s genome. However, this process is incomplete since cells cannot differentiate into three germ layers *in vivo*.

#### 3.2.3 Small Molecules Induction

Small molecules inhibit the expression of genes involved in cell differentiation and development. Small molecules, such as valproic acid, CHiR99021, sodium butyrate, vitamin C, parnate, 5-Azacytidine, and RG108, can replace factors from the core of the pluripotent network and importantly increase the efficiency of reprogramming. This method hopefully would be an alternate for the transcription factors.

#### 3.2.4 Transcription Factors

This method is used to generate iPSCs but has certain issues, such as low reprogramming efficiency and teratoma formation. iPSCs are reprogrammed from human somatic cells *via* ectopic expression of different transcription factors, but only four of them are sufficient to regulate the process: Oct4, Sox2, Klf4, and c-Myc (OSKM) [26]. Analyzing the transcriptional profile of the whole genome sequence indicated hundreds of pluripotency markers to be related to ESCs, but only three of them seemed to be essential regulators in early development and maintenance of ESCs identity [27]. The process starts with changes in the transcriptome and chromatin structure of differentiated cells in a pluripotent-like state. Changes in chromatin structure in somatic cells, including DNA methylation, histone modifications, and ATP-dependent chromatin remodeling, help the transcription factors to stick to the recognition sequence associated with pluripotency. Transcription factors tend to bind together to form an interconnected autoregulatory circuitry, trigger their own core promoter genes, and cooperate with other related genes. Interconnected auto-regulatory loop shows that Oct4 and Sox2 play a key role in the conservation of pluripotency especially in early embryo precursor cells [25, 28].

The exact procedure for using OSKM transcription factors to turn unipotent cells into pluripotent cells is still under debate. However, studies suggest that two transcriptional waves have a crucial role during pluripotency induction. In the first transcriptional wave, c-Myc binds to a large vicinity of the somatic genome with methylated H3K4me2 and H3K4me3, which mark the open chromatin. The second wave involves reprogramming of the cells; OSKM transcription factors access the enhancers and promoters of early pluripotency-related genes [29], and trigger transcription and expression of pluripotency. Activation of core pluripotency-related genes is the major characteristic of the subsequent reprogramming phase [25, 28].

Different ways to prepare iPSCs for therapeutic applications are mentioned below:

#### 3.2.5 Reprogramming by Integrative Viral Vector Transfer System

It provides temporal gene expression of the exogenous DNA sequence. The quality of iPSCs is partly impaired due to the inability to fully activate the expression of pluripotency endogenous genes [25, 30].

#### 3.2.6 Reprogramming by Integrative Non-viral Transfer System

This type is safer than the previous method. The first successful non-viral iPSCs were prepared from mature embryonic fibroblast cells transfected with two plasmid constructs; the first plasmid encodes for the c-Myc, while the second was a polycistronic vector encoding the four defined reprogramming factors [25, 31].

#### 3.2.7 Reprogramming by Non-integrative Non-viral Transfer System

To avoid using a vector, the pluripotency genes can be directly delivered into cytoplasmic RNA, episomal (self-replicating and selectable vectors), or polycistronic minicircle DNA non-viral vector systems. This strategy is easy to use but has low efficiency [25, 32].

#### 3.2.8 Reprogramming by Non-integrative Viral Transfer System

Although by employing this method, the obtained iPSCs did not exhibit any evidence of DNA exons in the host genome, the efficiency was limited to 0.001% [25].

## 4 OPCS AS A TREATMENT

### 4.1 Sources and *in vitro* Generation

Demyelination of intact axons leads to disorders of CNS, such as MS. OPCs are small cells with a bipolar or tripolar shape that are found in the white and gray matter of the CNS [33]. OPCs express markers, including A2B5, neural/glial antigen 2 (NG2), and platelet-derived growth factor receptor alpha (PDGFRα) [34]. They differentiate into OLs, which contribute to the remyelination of axons [35-38]. Remyelination by OPCs is deteriorated in demyelinating diseases [39].

Some OPCs persist in adults' brain and could differentiate into OLs to enhance remyelination at the injury site [40]. To treat demyelination disorders, transplantation of OPCs can be effective [34, 37, 40]. Various sources of OPC generation and implantation have been suggested. They can be obtained *in vitro* from ESCs, NSC, iPSCs, and bone marrow cells (BMs) [34, 38, 41, 42]. The application of NSC and ESC faces some limitations. NSCs have less potential for proliferation and differentiation than other cells. Moreover, ethical concerns and the risk of immune rejection due to their allogenic engraftment restrict the usage of ESCs and NSCs [38, 43]. While iPSCs application is considered an appropriate source without immune rejection and ethical issues, it may result in tumorigenesis [44].

Several protocols are utilized to generate OPCs as follows:

#### 4.1.1 ESCs-derived OPCs

Hu *et al.* described a protocol for hESCs-derived OPCs. The protocol is comprised of four parts. First, they induced neuroepithelial from hESCs under a lack of growth factors for 2 weeks. To generate OLIG2 progenitors in the second step, retinoic acid (RA), a caudalization factor, and sonic hedgehog (SHH), a ventralization morphogen, were applied. The progenitors were characterized by the expression of OLIG2. In the third step, the culture condition was changed by adding fibroblast growth factor2 (FGF 2) and removing RA. Hence, the OLIG2 progenitor cells express NKX2.2 (a transcription factor) known as pre-OPCs. Lastly, they cultured the pre-OPCs in a glia media comprising neurotrophin3 (NT3), PDGFR, cAMP, insulin-like growth factor-1 (IGF-1), biotin, and triiodothyronine (T3), to generate OPCs, which took 8 weeks. OPCs were identified by expressing NKX2.2, OLIG2, PDGFRα, SOX10, and NG2. The total population of OPCs was 80% in the 14^th^ week. The population of OLs differentiated from OPCs was 40% of the total cells. Most of the O4^+^ immature OLs were immature without myelin generation, but survived OLs expressed MBP to produce myelin sheaths [45]. Several studies report that RA and SHH promote the derivation of OPCs [46, 47].

#### 4.1.2 Mesenchymal Stem Cells (MSCs) Derived OPCs

Endometrial stromal cells (EnSCs) derived from human endometrium tissue could be a great source for neural cells. Ebrahimi *et al.* cultured EnSCs in a neuronal condition medium and induced OPCs by adding FGF2 and EGF. OPCs then expressed Sox10, Olig2, PDGFRα, and O4. Afterwards, they kept OPCs under treatment with PDGF, FGF, and T3, which down-regulated the expression of PDGFRα and led to immature OLs morphologies. EnSCs-derived OPCs expressed OL markers, including Sox10, Olig2, O4, and A2B5. However, the efficacy of OLs was not identified *in vivo* [48].

Zhang *et al.* described a protocol that induces OPCs from mouse bone marrow (BM) in a condition media consisting of T3, NT-3, IGF, and SHH. First, NSCs were generated from BM in a culture containing EGF and FGF2. BM-derived NSCs were transmitted to OPCs by PDGFα and bFGF. Besides, the presence of PDGFRα enhanced the cell population. This protocol acquired OLs within 40-50 days [34].

#### 4.1.3 Reprogramming Derived OPCs

Different protocols have been employed to generate OPCs from iPSCs [49]. Recently, cellular reprogramming by transcription factors (TFs) and chemicals has garnered attention as an appropriate strategy for iPSCs generation from somatic cells [42].

Douvaras *et al.* obtained iPSC from reprogrammed skin fibroblasts in primary progressive multiple sclerosis (PPMS) patients. NSCs PAX6^+^ were induced by dual-SMAD signaling inhibition in the culture condition. Then, adding RA and SHH promoted the differentiation of OLIG2 progenitor cells from NSCs by organizing the environment, like the spinal cord. On day 20, PDGF-AA, HGF (hepatocyte growth factor), NT3, and IGF-1 were added to the culture medium for late OPCs generation, which was detected by O4 live staining. Cells successfully expressed OLIG2, SOX10, and NG2. This protocol provides 44-70% O4^+^cells in 75 days compared to other protocols. About 34% of O4^+^OPCs were capable of generating MBP^+^ OLs *in vitro* [50]. Magnani *et al.* reported a protocol using spinal cord development signaling to induce OPCs. During the first 7 days, human iPSCs were neuralized under dual-SMAD inhibition. To induce OLIG2 progenitor cells, RA and SHH were supplied. Subsequently, the addition of PDGFα, IGF, FGF2, and T3 converted OLIG2 progenitor cells to PDGFRα-OPCs. Removal of these factors developed MBP^+^ OLs differentiation [51].

Yao *et al.* derived iPSC from the menstrual blood-derived stromal cell (MnSCs) of 4 secondary progressive multiple sclerosis (SPMS) patients through reprogramming factors. At first, iPSCs were cultured in a non-adherent condition to create embryoid bodies [52] without bFGF. After the addition of RA, colonies expressed PAX6, SOX1, and Tuj1, which are neural epithelial (NE) markers (63% of SPMS-iPSC colonies and 60% of control colonies) adding SHH-generated pre-OPCs expressing Olig2. Early OPCs expressing Sox9, NKX2.2, and Olig2 were found after RA withdrawal. OPCs expressing SOX10 and A2B5 were formed under a glial induction medium with NT3, PDGF-AA, and IGF-1 after 120 days. Expression of pre-OPC markers was close in SPMS and control lines. Approximately, 70% of cells were PAX6^+^ cells. In the early OPC stage, the expression of Olig2 was higher in the SPMS group rather than control (90% in the SPMS line and 80% in the control line). Hence, the authors demonstrated no significant difference between SPMS and control-derived cells. In addition, no distinction in survival was detected between both cell lines [53].

Najm *et al.* designed a protocol to induce PDGFRα-OPCs from hiPSCs. Sox10 and Olig2 TFs together were applied to speed up the procedure more than any single factor. Other studies reported a third factor, like Zfp536 or Nkx6.2, as efficient for converting fibroblasts into OPCs. To examine the myelination potency of OLs, induced OPCs were plated in co-culture with rat cortical neurons. After 14 days, 0.5% of rat neurons had axons positive for O4^+^OLs. Results showed 50% proportion of typical OLs to abnormal cells [36, 54].

Czepiel *et al.* patterned OPCs from mouse iPSCs. iPSCs from mouse embryonic fibroblasts (MEFs) were cultured in EB medium as EBs. After 8 days, EBs were cultured in a serum-free N2 medium to produce NSCs. NSCs derived from iPSCs were cultured in PDGF, FGF2, and epidermal growth factor (EGF). Eventually, NG2+OPCs were emerged. After T3 and NT-3 addition to the culture, OPCs started to differentiate into OLs. 6 days later, 18% of cells were MBP^+^ OLs. To investigate the myelination capacity, the iPSC-derived OPCs were cultured and differentiated into mature OLs able to myelinate the axons *in vitro* [49].

Terzic *et al.* derived 3 lines of iPSCs from mouse embryonic fibroblasts by using Oct4, Sox2, Klf4, and c-Myc. After 4 days, accumulation of miPSCs in serum-free medium and RA led to the formation of neural aggregates that expressed Pax6. Adding RA mimicked the spinal cord phenotype. To generate early OPCs expressing A2B5, Olig2, and PDGFRα^+^ on the 8^th^ day, purmorphamine (an agonist of smoothened receptors stimulating the hedgehog-signaling pathway) was added to RA, and the culture was changed to N-2 medium. On that point, early OPCs were cultured in an OPC medium with fibronectin and poly-L-ornithine. 7 days after, bipolar OPCs expressed Olig2 and PDGFRα^+^ emerged. During early OPCs differentiation, the expressions of Olig2, Olig1, PDGFRα, and NKX2.2 were raised. No expression of Oct4 and Nanog indicated the absence of pluripotent cells in the culture on the 25^th^ day. Three lines of miPSCs had the capacity to differentiate into OPCs. Two lines reached over 70% Olig2^+^ and A2B5^+^ cells, while the other line produced only 20%. In addition, data confirmed few numbers of GFAP^+^ astrocytes, which indicated the capacity of application of the OPC population. Finally, OPCs could differentiate into OLs after 3 weeks [40].

Tokumoto *et al.* compared the effectiveness of iPSCs in OPCs differentiation with ES cells. They applied mouse embryonic fibroblasts derived-iPSCs (MEF) and assessed the expression of Nanog (a stem cell marker) and rates of proliferation during 72 h. Both lines exhibited similar findings. For instance, cell clusters were plated in LIF-free ES medium and formed EBs that were same in shape and size. EBs were cultured in an ITS/fibronectin medium. Both iPSC and ESC-derived cells expressed Nestin. Nestin^+^ cells were plated in poly-L-ornithine/fibronectin slide flasks with growth factors. In the next 4 days, they were cultured in N2 plus/bFGF medium, then another 4 days in N2 plus/bFGF + EGF and N2 plus/bFGF + PDGF medium for the last 4 days, and A2B5^+^ OPCs were observed (15.1% for iPSC lines and 15.5% for ES lines). The culture was changed to T3 medium for terminal differentiation. O4-positive OLs were detected almost after a week. Despite 24% OLs from the ESC line, only 2.3% OLs were reported from iPSC lines [38].

Angelo *et al.* described a method for iPSC-derived OPCs generation. They used blood monocytes, MSCs, and respiratory fetal fibroblasts to generate iPSC lines. The transition of iPSCs to OPCs began by EBs formation in neurobasal media. EBS were cultured in media with FGF2 for 15 days. After 24 h, cells morphology changed to neural progenitors (NPs). Glial progenitors (GPs) appeared by adding EGF to the culture. GPs colonies differentiated into OPCs and astrocytes due to their media. 80% of cells expressed Nestin and A2B5 when FGF2 was removed and PDGF-AA was added at the NPs stage. At the GP stage, in addition to Nestin and A2B5, cells expressed NG2 and PDGFRα. In blood monocyte-derived iPSCs lines, 60% of cells were O1^+^OPCs. Although, in 2 other lines, O1 expression was lower, and Nestin expression was 60% [55].

Numasawa *et al.* established iPSCs from fibroblasts of Pelizaus-Merzbacher disease (PMD) patients and healthy individuals employing Sox2, Klf4, c-Myc, and Oct4. iPSC colonies formed EBs. To encourage NS/OPC differentiation, SB431542 (TGFβ receptor inhibitor), BIO (GSK3 inhibitor), and dorsomorphin were included during EB formation. PCR results demonstrated that Sox1 (a marker of NS/OPC) is substantially expressed in comparison to dual smad inhibition method. Meanwhile, RA and purmorphine were added to EBs for caudalization and ventralization, respectively. After that, EBs were cultured in the medium with differentiation factors to induce OL lineages. During differentiation, the expression of pluripotency markers like Nanog diminished. 95.1% and 90.5% of PMD 1 and 2 cell lines were PDGFRα^+^ and OLIG2+OPCS, while 86.3% of control cells differentiated into OPCs after 55-70 days. The percentage of immature OLs was reported to be 93.8% in both PMD groups and 77.8% in the control line. After 95 days, 74.9% of control colonies differentiated into mature Ols; meanwhile, 93.8% of PMD1 lines and 89.2% of PMD2 lines were successfully transmitted *in vitro* [56].

Morales *et al.* derived iPSC from peripheral blood mononuclear cells (PBMCs) of progressive and stable MS patients. They produced OPCs by Douvaras and Fossati protocol. O4^+^ and MBP^+^ OLs from iPSC-derived OPCs emerged after 85 days. To assess the myelination capacity, two approaches were engaged. In the first one, OLs and neurons were plated in two chambers. After 30 days, data showed myelinated axons in all cell lines. Results demonstrated MBP^+^ OLs to be differentiated from iPSC-derived OPCs of MS patients. Morales and colleagues studied the effects of inflammation on iPSCs differentiation. They examined different markers from the 36^th^ day between treated and untreated cells with IFNγ in cultures. Suppression of OL development during early treatment with a low dose of IFNγ was higher than dilatory treatment on the 60^th^ day. In untreated groups, expression of PDGFRα^+^ was down-regulated; thus, OPCs were differentiated into OLs. Alleviation of inflammation might enhance the efficacy of OPC differentiation [57].

Fig. (**1**) shows *in vitro* generation of OPCs from iPSCs.

#### 4.1.4 OPCs Derived from Primary Cultures

One of the methods for the derivation of OPCs is purification from primary cultures. Several protocols have been developed to isolate OPCs from different parts of rodents’ brains, including shaking methods, immunopanning, and fluorescence-activated cell sorting (FACS) [58].

In the shake-off technique, cells dissociated by centrifuge are cultured in a medium with fetal bovine serum (FBS), leading to the proliferation of OPCs. However, FBS could stimulate bone morphogenetic protein (BMP) signaling and cause differentiation into astrocytes. Also, OPCs would not be dissociated from astrocyte layers [59, 60]. Dugas and Emery described a protocol to purify OPCs from rodent brain by immunopanning technique that depletes undesirable cells by negative selection and isolate OPCs through positive selection and enzymes [61]. Immunopanning provides an opportunity to extract OPCs from different brain parts at various developmental stages. Immunipanning can purify a higher percentage of OPCs rather than shake-off [58]. Despite these advantages, it is an expensive method, and there are no appropriate antibodies for selection [62]. Another aspect of the isolation of OPCs from various regions of CNS is different characterization, which shows a distinct tendency to differentiate into OLs. For example, the generation of OLs in the cortex takes more time than the optic nerve [63].

Table **1** describes some advantages and limitations of OPCs derived from different sources.

### 4.2 Migration and Differentiation

Lack of OPCs in SPMS lesions is an impediment in treatment associated with impaired migration. The capacity of differentiation and migration of iPSCs from SPMS-affected donors is similar to age-matched controls (CT), which is validated *in vitro*. Although the secreted proteins from SPMS-OPC are different from CT with the exogenous addition of specific components to their conditioned media [64], their capacity for migration is reinforced [53].

Since mature cells are less motile, maturation appears to be contrary to migration. Nevertheless, chemokine (C-X-C motif) ligand 1 **(**CXCL1) is one of the factors enhancing differentiation and proliferation as well as migration. The effect of factors may rely on timing, dosage, expression of the receptors, and other components. As a result, the development of strategies for using these agents holds promise for future treatment of demyelinating diseases [65]. Semaphorins are a group of secretory and membrane proteins that act as chemotactic factors and directors in axons for oligodendroglia cells during development of the nervous system [65]. In addition, they have a major role in the damage repair phase of the CNS. SEMA3F increases OPCs migration *in vitro*, which is secreted by astrocytes, microglia, and OLs. Compston *et al.* showed that OPC migration is reduced in Sprague-Dawley rats during inhibition of SEMA3F receptor expression (*e.g*., neuropilin-2 (NRP-2) and plexin A3) by siRNA transfection [3]. Thus, NRP-2 and plexin A3 are associated with OPC migration. NRP-2 is more effective than plexin A3, and the function of plexin A3 in OPC migration is unclear in different lesion types. In chronic active lesions (which are less inflammatory), sema3A is expressed more than sema3F. In contrast, in acute active lesions (which are more inflammatory), cells express more sema3F than sema3A. The high level of sema3A expression may recover remyelination. On the other hand, the lower expression of sema3F tends to minimize it. Therefore, the amount of these factors significantly impacts remyelination [5].

Netrin-1 is another secretory protein regulating OPCs migration. It is present in chronic MS plaques [66], which impedes OPCs recruitment and makes it a compelling chemorepellent for migration. Netrin-1 prevents axonal growth and reduces CNS regeneration [67]. Nevertheless, it increases the number of blood vessels in lesion areas [68]. Despite that, in mature and healthy CNS, Netrin-1 and its fragments may have the desired impact by repelling cell migration and axon growth due to preventing aberrant sprouting [5].

Overexpression of the polysialylation enzyme sialyl transferase X (STX) in iPSC-derived OPCs improves the migration of OPCs, which is validated in both *in vivo* and *in vitro* studies. In these cells, polysialic acid-neuronal cell adhesion molecules (PSA-NCAMs) increase, which is essential for OPCs migration. Though, PSA-NCAMs have to be down-regulated for the beginning of myelination [69].

OLs are responsible for remyelination in the brain [70, 71]. OPCs present in the demyelination site of MS patients, but eventually, differentiation to OLs is deteriorated [42]. During OPCs differentiation, their morphology changes and new cell markers are obtained. At first, OPCs transmit to pre-OLs, and then differentiate to mature OLs that express MBP, myelin-associated glycoprotein (MAG), transmembrane proteolipid protein (PLP), and galactocerebroside (Ga1C) [72, 73]. OPCs can differentiate into OLs and astrocytes by different factors and signals [74], as we have mentioned below:

#### 4.2.1 TFs

Different TFs contribute to this mechanism. Helix-loop-helix (HLH) family, like OL transcription factors OLIG1, OLIG2, and achaete-scute homolog 1 (ASCL1), induces OPC differentiation into OLs. However, OLIG1 is less crucial in OPC differentiation. Sex-determining region Y-box (Sox) 10 is involved in terminal differentiation. In reverse, Sox 5 and Sox 6 compete with Sox 10 and hinder differentiation. Although Sox 17 has a negative role in OPC differentiation, Nkx2.2, Nkx6.2, and Smad-interacting protein 1 (Sip1) enhance it. Yin yang 1 (YY1) prevents inhibitor proteins, such as Id4, and promotes oligodendrocyte differentiation. Conversely, hairy and enhancer-of-split homologs (HES) 1 and HES 5, inhibitors of DNA binding (Id) 2, and Id4 prevent differentiation of OPCs [72, 75-78].

#### 4.2.2 Epigenetic Factors

In addition to TFs, epigenetic processes, like microRNAs, DNA methylation, and histone modification, regulate OPC differentiation into OLs. microRNAs (miR) are small non-coding RNAs. miR-219, miR-338, and miR-146 have a key role in the differentiation of OPCs. Down-regulated amounts of these microRNAs lead to halted OPC differentiation in MS. Contrary, miR-9 is reduced during differentiation to OLs [75, 77-79]. DNA methylation by DNA methyltransferases (DNMTs) also impacts OPCs’ differentiation. DNA methylation and DNMT1 inhibit the OPC differentiation and diminish OLs number [77].

Besides the above mechanisms, reduced voltage-gated potassium and sodium are relevant to OPC differentiation [80, 81]. Chanoumidou *et al.* demonstrated that voltage-gated chloride channel (CLC)-2 induces OPC differentiation by controlling TFs [80].

Two strategies are available to surge OL production: 1) The gathering of endogenous OPCs or 2) their transplantation to form OLs (Lopez Juarez *et al.*, [74] 2016). Some studies have grafted iPSC-derived OPC *in vivo* to ameliorate remyelination, as described below.

#### 4.2.3 MS Models

For the first time, ESC-derived OPC-like cells were transplanted into a model of myelin-deficient rats [82].

Douvaras *et al.* implanted iPSC-derived OPCs that were generated from skin fibroblast of PPMS patients into the forebrain of immunocompromised shiverer mice. After 12-16 weeks, mice were sacrificed. Implanted cells were distributed in the white matter of the forebrain and corpus callosum. Over 80% of cells expressed OLIG2 in the corpus callosum and differentiated into OL lines. At the 16^th^ week, 31% of axons were myelinated, and OPCs migrated through the cortex. Although, few numbers of GFAP^+^astrocytes differentiated from O4 cells in the SVZ. Furthermore, results showed the efficacy of PPMS-derived OPCs *in vivo* to be the same as healthy derived cells [50].

Thiruvalluvan *et al.* assessed the effectiveness of hiPSC-derived OPC in the experimental autoimmune encephalomyelitis (EAE) model of marmosets. hiPSCs were derived from four healthy human skin fibroblasts. First, they examined the functionality of hiPSC-derived OPC in the EAE model of mice compared to the control group. HiPSC-derived OPCs labeled with GFP were implanted in the median line in the cortex above the corpus callosum. Also, the marmosets received cyclosporine daily. The animals were sacrificed at 20, 30, and 40 days after transplantation. Implanted OPCs survived 40 days. They migrate to demyelinated sites in the corpus callosum and differentiate into myelinating cells as well. 60% of implanted cells in one area of the corpus callosum were MBP^+^cells on their 40^th^ day that could myelinate axons. Few numbers of GFAP^+^cells were detected. Although the clinical score was not significantly reduced, which is probably related to limited diffusion [43].

#### 4.2.4 Other Models

Czepiel *et al.* implanted iPSC-derived OPCs in the cuprizone mouse model. The cuprizone diet led to axon demyelination in the corpus callosum. After 12 weeks, the first and second groups of mice received a sorted and unsorted suspension of iPSC-derived OPC, respectively. The third group acquired an unsorted suspension of undifferentiated iPS cells. After 2 weeks, undifferentiated iPS cells suspension formed a teratoma. 80% of implanted cells were lost. After 4 weeks, survived sorted iPS-derived OPCs expressed MBP and supplied remyelination of the axons in the corpus callosum [49].

Wang *et al.* evaluated the potential of hiPSC-derived OPCs transplantation in a congenital hypomyelination model of the mouse. They obtained iPSCs from keratinocytes and fibroblasts. After inducing OPCs *in vitro*, they transplanted 100,000 hiPSC-derived OPCs into the corpus callosum of newborn shiverer mice. 4.5 months later, mice were sacrificed. All of the lines demonstrated myelinated corpus callosum. 78.7% of transplanted cells remained OLIG2^+^ progenitors and oligodendroglia. Others differentiated to GFAP^+^ astroglia. To assess the efficacy of grafts and survivability of mice, at 20 weeks of age, 19 shiverer mice of the unimplanted group died earlier. 19 mice of the implanted group survived over 5 months and showed myelination of the cerebellum and the brain stem. Although, the assessment of 5 mice after 6 months showed no sign of tumorigenesis, it is still the main concern and needs more investigation [83].

Pouya *et al.* induced demyelination in optic chiasma of rats by lysolecithin. They implanted iPSC-derived OPC in rats. 8 weeks later, most of the implanted cells differentiated to OLs expressing MBP. Luxol fast blue staining revealed remyelination of axons. Few numbers of cells differentiated into GFAP^+^astrocytes. Recovery in the engrafted group was noticed a week after transplantation. Examination after eight weeks indicated no undifferentiated iPSCs and teratoma formation [84].

Kawabata *et al.* transplanted iPSC-derived OPCs in the spinal cord injury model of mice [85]. 33.6% of implanted cells in the lesion area differentiated into APC^+^ OLs. 39% of implanted migrated cells transmitted to APC^+^OLs, while 33% differentiated into GFAP^+^astrocytes. At the 12th week, 35.5% of engrafted cells differentiated to adenomatous polyposis coli CC-1 (APC) ^+^ OLs and led to the build up of myelin sheaths, leading to developed function. Also, immunostaining illustrated the integration of implanted-derived cells into host synapses. No tumorigenesis was observed after 12 weeks [86].

To conclude, iPSC-derived OPCs have been applied to different models of hypomyelination. Despite incomplete differentiation of all implanted cells into OLs, they could migrate and remyelinate injury sites comparatively. Fig. (**2**) summarizes the impacts of iPSC-derived OPCs on myelination.

## CONCLUSION

iPSCs are applied to generate OPCs that differentiate into OLs and form myelin. iPSC-derived OPCs benefit in comparison to other sources, like ESCs and NSCs. iPSC-derived cells could be utilized as an autologous source of stem cells that minimize the risk of cell rejection. However, there are some challenges in the application of iPSC-derived cells.

Generation of iPSC-derived OPCs and OLs takes a long time *in vitro*, as discussed in this review. On the other hand, the purity of OPCs is not high and the duration of OL differentiation is long [69].

Several animal studies have determined the efficacy of iPSC-derived OPCs transplantation in hypomyelination models. However, concerns exist about transplanted cells. For instance, OPCs are sensitive to oxidative stress. Also, their survival and migration are not satisfactory [87]. Therefore, the percentage of differentiation is not enough. The risk of tumorigenesis is still unsolved since undifferentiated transplanted cells could form teratoma [87-89]. However, the threat of genetic and epigenetic transformation of iPSCs during reprogramming may affect the efficacy of iPSCs-derived cells [89, 90]. Despite the risks of iPSC-derived OPCs, they need more studies in primate models and humans, but they could be a suitable source for future stem cell therapy in demyelination diseases, like MS.

## Figures and Tables

**Fig. (1):**
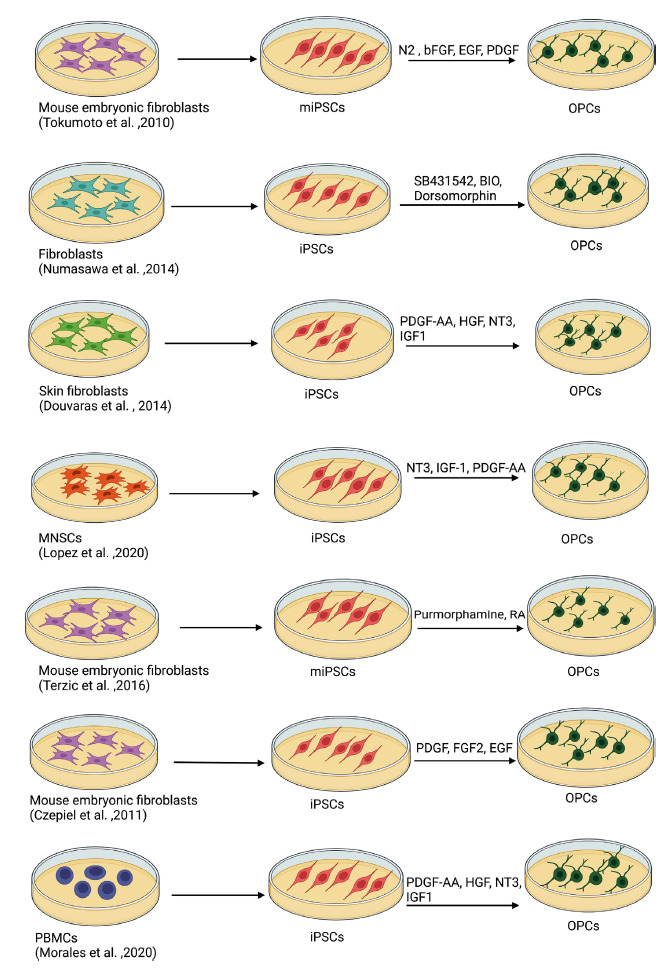
*In vitro* generation of iPSC-derived OPCs from differentiated cells. **Abbreviations**: iPSC, induced pluripotent stem cell; OPC, oligodendrocyte progenitor cell.

**Fig. (2):**
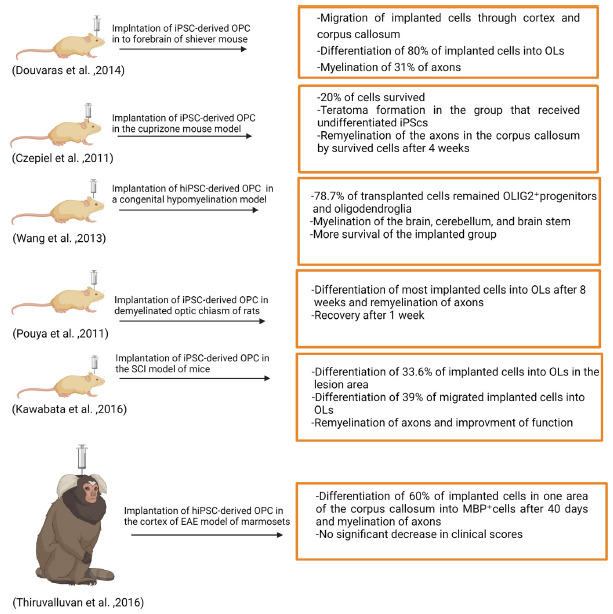
*In vivo* generation of OLs from implanted iPSC-derived OPCs. **Abbreviations**: OL, oligodendrocyte; iPSC, induced pluripotent stem cell; OPC, oligodendrocyte progenitor cell.

**Table 1:** The advantages and limitations of OPCs derived from different sources.

**Sources of OPCs**	**Strengths**	**Limitations**
Primary cultures	Without risk of immune rejection Differentiation into oligodendrocytes (OLs)	Different behavior of oligodendrocyte precursor cells (OPCs) from various origins in the central nervous system (CNS) Long preparation time Much expense for immunopanning Adverse effects of culture supplements Dependency of the proliferation rate to the age of samples
ESCs derived OPCs	High percentage of differentiation into OLs	Ethical restrictions Immune rejection Short supply
NSCs derived OPCs	Effective differentiation into OLs	Ethical restrictions Immune rejection Difficult accesses to neural stem cells (NSCs) in the brain Sensitive to inflammation
iPSCs derived OPCs	An autologous source without the risk of rejection Improvement of remyelination in *in vivo*	Risk of tumorigenesis Long duration of *in vitro* generation Less differentiation into OLs

## LIST OF ABBREVIATIONS

ASCs: Adult Stem Cells
BMP: Bone Morphogenetic Protein
BMs: Bone Marrow Cells
CNS: Central Nervous System
EGF: Epidermal Growth Factor
hESCs: Human Embryonic Stem Cells
HSCs: Hematopoietic Stem Cells
iPSCs: Induced Pluripotent Stem Cells
MnSCs: Menstrual Blood-derived Stromal Cell
MS: Multiple Sclerosis
MSCs: Mesenchymal Stem Cells
NSCs: Neural Stem Cells
OLs: Oligodendrocytes
OPCs: Oligodendrocyte Progenitor Cells
